# Robust Non-Rigid Feature Matching for Image Registration Using Geometry Preserving

**DOI:** 10.3390/s19122729

**Published:** 2019-06-18

**Authors:** Hao Zhu, Ke Zou, Yongfu Li, Ming Cen, Lyudmila Mihaylova

**Affiliations:** 1 Key Laboratory of Intelligent Air-Ground Cooperative Control for Universities in Chongqing, and Automotive Electronics and Embedded System Engineering Research Center, College of Automation, Chongqing University of Posts and Telecommunications, Chongqing 400065, China; kezou18@163.com (K.Z.); liyongfu@cqupt.edu.cn (Y.L.); cenming@cqupt.edu.cn (M.C.); 2 Department of Automatic Control and Systems Engineering, University of Sheffield, Mappin Street, Sheffield S1 3JD, UK; L.S.Mihaylova@sheffield.ac.uk

**Keywords:** image registration, non-rigid feature matching, local structure descriptor, Gaussian mixture model

## Abstract

In this paper, a robust non-rigid feature matching approach for image registration with geometry constraints is proposed. The non-rigid feature matching approach is formulated as a maximum likelihood (ML) estimation problem. The feature points of one image are represented by Gaussian mixture model (GMM) centroids, and are fitted to the feature points of the other image by moving coherently to encode the global structure. To preserve the local geometry of these feature points, two local structure descriptors of the connectivity matrix and Laplacian coordinate are constructed. The expectation maximization (EM) algorithm is applied to solve this ML problem. Experimental results demonstrate that the proposed approach has better performance than current state-of-the-art methods.

## 1. Introduction

Image registration is a fundamental task in many fields, such as computer vision, robotics, medical image processing, and remote sensing [1,2,3,4]. The main purpose of image registration is to align two or more images of the same scene taken from different viewpoints, at different times, and/or by different sensors.

Many algorithms have been developed for image registration. It can be roughly divided into area-based and feature-based methods. Area-based methods match image intensity values directly. They mainly include the cross-correlation (CC) methods, the Fourier methods, and mutual information (MI) methods. The normalized CC method is a classic in the area-based methods [5]. The similarity of window pairs from two images are computed and the maximum is considered as a correspondence. A  method based on wavelet decomposition and correlation is proposed for image registration [6]. The  Fourier methods find the Fourier representation of the images and a subpixel phase correlation with Gaussian mixture model (GMM) is used to register the images [7]. The MI method provides a measure of dependence between two images, and a deterministic explanation for MI-based image registration is proposed in [8].

Feature-based methods extract the salient structures, i.e., features, from the images. The extracted features are called control points [9], and the feature-based methods are considered as point set registration. The traditional for feature-based image registration approach uses a two-step strategy. In the first step, the distances of feature points from local descriptors, such as scale-invariant Fourier transform (SIFT), speeded-up robust features (SURF), or shape context (SC), are computed to obtain putative correspondences. The putative correspondences may contain some true matches, false matches, and outliers. The true matches from the putative correspondences are then distinguished by some geometrical constraint methods. In [10], an improved random sample consensus (RANSAC) algorithm is proposed to get correct matches, and a common visual pattern is detected for feature matching in pairwise images [11]. In [12], the progressive vector field consensus (VFC) is used to establish the feature points correspondences between images.

The feature-based image registration is formulated to estimate the correspondence matrix and the transformation function. This strategy can be categorized as distance-based methods and probability-based methods [13]. Two-step schemes are employed in distance-based methods. The two point sets distance with the computed correspondence is minimized to estimate the transformation. Widely used methods include the iterative closest point (ICP) algorithm [14] and its variants [15]. Using least squares (LS) minimization, the closest point is considered as the correspondence and the transformation is estimated in the ICP algorithm. In [16], the similarity measures for matching and registration are used as thin-plate spline (TPS) functions in the non-rigid case. In [17], a  Havrda–Charvat–Tsallis entropy is proposed to register medical images.

For the probability-based methods, feature points are represented as probability density functions. These methods have attracted considerable attention [18,19,20,21,22,23,24]. In the CPD method [18], one point set is represented by Gaussian mixture model (GMM) centroids, and the other point set is fitted to the first point set by moving coherently. In [19], the point sets from medical images are represented as a GMM, the Jensen–Shannon (JS) divergence between these Gaussian densities is minimized for image registration. In [20], both point sets are modeled as GMM, the Euclidean distance between these GMMs is minimized to achieve registration. The above-mentioned methods only consider the global structure between two point sets. In [21], a local descriptor, which is called as a local linear embedding (LLE), is proposed to preserve local structure between two point sets. In [22], a locally linear transforming (LLT), which is similar to LLE, is used to match feature points for remote sensing image. In [24], a  dual-feature based point set registration with global-local structural preservation is  proposed.

In this paper, we propose a novel GMM-based non-rigid point set registration method to perform image feature matching with structure constraints. The feature points of one image are represented by GMM centroids, and the feature points of the other image are considered as data points. The GMM centroids are moved coherently to fit the data points using a non-rigid transformation by maximizing the likelihood, which encodes the global structure of the pairwise image feature points. For a smooth non-rigid transformation, the coherent constraint is applied by regularization of the displacement field. The shape context is used for the membership probabilities initialization. Furthermore, two local structure descriptors are constructed. The first descriptor is inspired by the idea that the local neighbors in the point set could be preserved after the non-rigid transformation. The Laplacian coordinate is used in the second descriptor to keep the size of the neighborhood structure. Therefore, the objective function is composed of a global distance item, non-rigid transformation constraint item, and two local structure constraint items. An expectation-maximization (EM) algorithm is then applied to perform the maximum likelihood (ML) optimization.

The contribution of this paper can be summarized as follows. (1) Two local structure descriptors of the connectivity matrix and Laplacian coordinates are constructed to preserve the local structure of feature points. The connectivity matrix-based constraint is proposed to retain the local neighborhood relationship, and the Laplacian coordinate-based constraint is employed to encode the local neighborhood scale. The proposed method preserves the local structure in terms of the neighborhood relationship and neighborhood scale, and it is more flexible. (2) The likelihood of image registration is formulated and the EM algorithm is proposed for feature-based image registration.

The paper is organized as follows. The problem of pairwise image registration is formulated in Section 2. The unknown parameters of the proposed method are estimated by the EM algorithm in Section 3. In Section 4, experiments are carried out to validate the performance of the proposed method. Finally, conclusions are given in Section 5.

## 2. Problem Formulation

Without loss of generality, the extracted feature points from pairwise images are represented as $X={\left\{x_{i}\left|x_{i}\in {ℜ}^{D}\right.\right\}}_{i=1}^{N}$ and $Y={\left\{y_{j}\left|y_{j}\in {ℜ}^{D}\right.\right\}}_{j=1}^{M}$, respectively, where *D* denotes the dimension of the feature points. Our goal is to find a suitable transformation and to establish the correct correspondence between X and Y. We set the non-rigid transformation of point set X as the GMM centroids and the other point set Y as the data point generated by the GMM. That is,

(1) $$p\left(Y\right)=\prod_{j=1}^{M}\sum_{i=1}^{N}\pi_{ji}N\left(y_{j}\left|f\left(x_{i}\right),\sigma^{2}I_{D}\right.\right)$$

where N denotes the Gaussian distribution, *f* means the non-rigid transformation, $\sigma^{2}$ represents the equal isotropic covariances, and I is the identity matrix. We introduce an indicator $Z=[z_{1},z_{2},\ldots ,z_{M}]$, where $z_{j}$ is a 1×M binary vector with elements $z_{ji}$ for i=1,2,…N. The $z_{ji}$ satisfy $z_{ji}\in \left\{0,1\right\}$ and $\sum_{i}z_{ji}=1$. It means that only one element in vector $z_{j}$ is equal to 1 and all the other elements in vector $z_{j}$ are equal to 0. We have

(2) $$p\left(z_{j}\left|\pi \right.\right)=\prod_{i=1}^{N}{\pi_{ji}}^{z_{ji}}$$

(3) $$p\left(y_{j}\left|x_{i},\sigma^{2},z_{j}\right.\right)=\prod_{i=1}^{N}N{\left(y_{j}\left|f\left(x_{i}\right),\sigma^{2}I_{D}\right.\right)}^{z_{ji}}$$

where $\pi ={\left\{\pi_{ji}\right\}}_{i=1:N}^{j=1:M}$. To account for outliers in Y, a uniform component $p\left(y_{j}\left|N+1\right.\right)=\frac{1}{M}$ with weight *w* is added to the mixture model, where *w* is a weight of the uniform distribution for noise and outlier. Assuming independent and identically distributed data in Y, we have

(4) $$p\left(y_{j}\right)=w\frac{1}{M}+\left(1-w\right)\sum_{i=1}^{N}\pi_{ji}N\left(y_{j}\left|f\left(x_{i}\right),\sigma^{2}I_{D}\right.\right)$$

The negative log-likelihood function of Equation (1) can be represented as:

(5) $$\begin{array}{cc} Q & =\sum_{j=1}^{M}\sum_{i=1}^{N}q\left(z_{ji}\right)\frac{{∥y_{j}-f\left(x_{i}\right)∥}^{2}}{2\sigma^{2}}\\ & +\frac{D}{2}\log\left(\sigma^{2}\right)\sum_{j=1}^{N}\sum_{i=1}^{N}q(z_{ji}) \end{array}$$

where $q(z_{ji})$ is the posterior probability.

The non-rigid transformation *f* is chosen as a displacement function [18]

(6) f(X)=X+GW

where G denotes a N×N dimensional Gaussian kernel matrix with element $g_{ij}=\exp\left(-\frac{1}{2}{∥\frac{x_{i}-x_{j}}{\tau }∥}^{2}\right)$, τ denotes the width of the Gaussian kernel, and W is a N×D dimensional weight matrix of the Gaussian kernel. In order to enforce the motion coherence for preserving the global topology, the  constraint on the weight matrix W given as reference is used [18]

(7) $$E_{w}\left(W\right)=\mathrm{Tr}\left(W^{T}\mathrm{GW}\right)$$

where Tr denotes the trace of matrix and $W^{T}$ is the transposition of matrix W. The objective function of feature-based image registration can then be written as

(8) $$\begin{array}{cc} Q & =\sum_{j=1}^{M}\sum_{i=1}^{N}q\left(z_{ji}\right)\frac{{∥y_{j}-f\left(x_{i}\right)∥}^{2}}{2\sigma^{2}}\\ & +\frac{D}{2}\log\left(\sigma^{2}\right)\sum_{j=1}^{N}\sum_{i=1}^{N}q(z_{ji})+\alpha \mathrm{Tr}\left(W^{T}\mathrm{GW}\right) \end{array}$$

where α is the trade-off parameter.

In Equation (1), the membership probability $\pi_{ji}$ is chosen in advance. Some methods set the membership probability to be equal [18,25,26,27]. For robustness, the feature of local shape is proposed here for membership probability initialization. In the 2D case, the shape context (SC) [28] is used as feature descriptor and the Hungarian method is used to perform point matching. In the 3D case, the fast point feature histograms (FPFH) [29] and a sample consensus initial alignment method are used for matching.

Then, two local structure descriptors are constructed to preserve the local structure of point set X. By computing the Euclidean distance between each point and its neighbors in X, the *K* nearest neighbors of each point in X can be obtained. Each point in X can be represented as a weighted linear combination of its *K* nearest neighbors. Let $L={\left\{L_{ij}\right\}}_{i=1:N}^{j=1:N}$ be an N×N weighted matrix. If a point $x_{j}$ does not belong to the *K* nearest neighbors of a point $x_{i}$, then $L_{ij}$ is set as 0. The matrix L can be obtained by minimizing the cost function:

(9) $$e\left(L\right)=\sum_{i=1}^{N}{∥x_{i}-\sum_{j=1}^{N}L_{ij}x_{j}∥}^{2}$$

where the sum of each of row of L is equal to 1. After the non-rigid transformation, the local structure can be preserved by minimizing the transformed cost function

(10) $$\begin{array}{c} E_{l}\left(L\right)\\ =\sum_{j=1}^{M}{\sum_{i=1}^{N}q\left(z_{ji}\right)∥f\left(x_{i}\right)-\sum_{j=1}^{N}L_{ij}f\left(x_{j}\right)∥}^{2}\\ =\sum_{j=1}^{M}{\sum_{i=1}^{N}q\left(z_{ji}\right)∥x_{i}+G\left(i,.\right)W-\sum_{j=1}^{N}L_{ij}\left(x_{i}+G\left(i,.\right)W\right)∥}^{2} \end{array}$$

where G(i,.) means the $i^{th}$ row of G. The objective function of feature-based image registration in Equation (8) can be expressed as:

(11) $$\begin{array}{c} Q_{1}\\ =Q+\beta E_{l}\left(L\right)\\ =\sum_{j=1}^{M}\sum_{i=1}^{N}q\left(z_{ji}\right)\frac{{∥y_{j}-f\left(x_{i}\right)∥}^{2}}{2\sigma^{2}}\\ +\frac{D}{2}\log\left(\sigma^{2}\right)\sum_{j=1}^{M}\sum_{i=1}^{N}q(z_{ji})+\alpha \mathrm{Tr}\left(W^{T}\mathrm{GW}\right)\\ +\beta \sum_{j=1}^{M}{\sum_{i=1}^{N}q\left(z_{ji}\right)∥x_{i}+G\left(i,.\right)W-\sum_{j=1}^{N}L_{ij}\left(x_{i}+G\left(i,.\right)W\right)∥}^{2} \end{array}$$

where β is the trade-off parameter. For the second structure descriptor, the Laplacian coordinate is proposed to preserve the size of neighborhood structure. For a graph (V,E), where V and E is the set of vertices and the set of edges in the graph, respectively, the Laplacian coordinate is expressed as

(12) $$J\left(v_{i}\right)=\sum_{(i,j)\in E}h_{ij}\left(v_{i}-v_{j}\right)$$

where (i,j) means the edge between vertices $v_{i}$ and vertices $v_{j}$ and $h_{ij}$ is the weight coefficient. The graph of point set *X* is constructed as follows: the vertex set is set as $V={\left\{x_{i}\right\}}_{i=1:N}$ and the edges $\left(x_{i},x_{j}\right)$ if and only if ${∥x_{i}-x_{j}∥}^{2}<\varepsilon$, where ε is the threshold parameter to construct the neighborhood graph. $h_{ij}$ is chosen as

(13) $$h_{ij}=e^{-\frac{1}{\varepsilon }{∥x_{i}-x_{j}∥}^{2}}$$

After the non-rigid transformation, the Laplacian regularization can be preserved by minimizing the following term:

(14) $$\begin{array}{c} E_{h}\left(h\right)=\sum_{j=1}^{M}\sum_{i=1}^{N}q\left(z_{ji}\right){∥J\left(x_{i}\right)-J\left(f\left(x_{j}\right)\right)∥}^{2}\\ =\sum_{j=1}^{M}\sum_{i=1}^{N}q\left(z_{ji}\right){∥J\left(x_{i}\right)-J\left(x_{i}+G\left(i,.\right)W\right)∥}^{2} \end{array}$$

The objective function of the feature-based image registration in Equation (11) can be updated as:

(15) $$\begin{array}{c} Q_{2}\\ =Q_{1}+\gamma E_{h}\left(h\right)\\ =\sum_{j=1}^{M}\sum_{i=1}^{N}q\left(z_{ji}\right)\frac{{∥y_{j}-f\left(x_{i}\right)∥}^{2}}{2\sigma^{2}}\\ +\frac{D}{2}\log\left(\sigma^{2}\right)\sum_{j=1}^{M}\sum_{i=1}^{N}q(z_{ji})+\alpha \mathrm{Tr}\left(W^{T}\mathrm{GW}\right)\\ +\beta \sum_{j=1}^{M}{\sum_{i=1}^{N}q\left(z_{ji}\right)∥x_{i}+G\left(i,.\right)W-\sum_{j=1}^{N}L_{ij}\left(x_{i}+G\left(i,.\right)W\right)∥}^{2}\\ +\gamma \sum_{j=1}^{M}\sum_{i=1}^{N}q\left(z_{ji}\right){∥J\left(x_{i}\right)-J\left(x_{i}+G\left(i,.\right)W\right)∥}^{2} \end{array}$$

where γ is the trade-off parameter.

## 3. EM for the Proposed Method

In order to estimate the $\Theta =[W,\sigma^{2},Z]$, the EM algorithm is proposed. There are two steps in the EM algorithm.

(1). $E_{L}\left(\Theta ,\Theta^{\left(m\right)}\right)=Q_{2}$
(2). $\Theta^{\left(m+1\right)}=\max E_{L}\left(\Theta ,\Theta^{\left(m\right)}\right)$

where *m* refers to the *m*th iteration. By iterating these two steps, the parameters Θ are determined while the likelihood function can also be increased.

### 3.1. E-Step

We use $q(z_{ji})$ to denote $p(z_{ji}=1\left|Y,X\right.)$, which can be found using the Bayes’ theorem

(16) $$q\left(z_{ji}\right)=\frac{\pi_{ji}N\left(y_{j}\left|f\left(x_{i}\right),\sigma^{2}I_{D}\right.\right)}{\sum_{i}\pi_{ji}N\left(y_{j}\left|f\left(x_{i}\right),\sigma^{2}I_{D}\right.\right)+\frac{w}{1-w}\times \frac{N}{M}}$$

### 3.2. M-Step

The $E_{L}\left(\Theta ,\Theta^{\left(m\right)}\right)$ can be rewritten as

(17) $$\begin{array}{c} E_{L}\left(\Theta ,\Theta^{\left(m\right)}\right)\\ =\frac{1}{2\sigma^{2}}\left\{\mathrm{Tr}\left(Yd(P1)Y\right)-2\mathrm{Tr}\left({\left(P^{T}Y\right)}^{T}\left(X+\mathrm{GW}\right)\right)\right.\\ \left.+\mathrm{Tr}\left({\left(X+\mathrm{GW}\right)}^{T}d\left(P^{T}1\right)\left(X+\mathrm{GW}\right)\right)\right\}\\ +\frac{D}{2}\log\left(\sigma^{2}\right)\sum_{j=1}^{M}\sum_{i=1}^{N}q(z_{ji})+\alpha \mathrm{Tr}\left(W^{T}\mathrm{GW}\right)\\ +\beta \mathrm{Tr}\left(X^{T}\mathrm{BX}\right)+2\beta \mathrm{Tr}\left(X^{T}\mathrm{BGW}\right)\\ +\beta \mathrm{Tr}\left(W^{T}\mathrm{GBGW}\right)\\ +\gamma \mathrm{Tr}\left(W^{T}GS^{T}d\left(P^{T}1\right)\mathrm{SGW}\right) \end{array}$$

where d(.) indicates diagonal matrix, P is the M×N matrix with element $q(z_{ji})$, $B={\left(I-L\right)}^{T}d\left(P^{T}1\right)\left(I-L\right)$, **1** is the all-one column vector, I is the identity matrix, **S** denotes the N×N Laplacian matrix, S=C−H, **H** is the N×N adjacency matrix with element $h_{ij}$, and C is the diagonal matrix whose i-th entry is the sum of ${\left(h_{ij}\right)}_{j=1,2,\ldots N}$.

The estimates of $\sigma_{k}^{2}$ and W are updated iteratively by taking the corresponding partial derivative of the expected log likelihood. That is,

(18) $$\begin{array}{c} \frac{\partial E_{L}\left(\Theta ,\Theta^{\left(m\right)}\right)}{\partial \sigma^{2}}\\ =-\frac{1}{2\sigma^{4}}\left\{\mathrm{Tr}\left(Yd(P1)Y\right)-2\mathrm{Tr}\left({\left(P^{T}Y\right)}^{T}\left(X+\mathrm{GW}\right)\right)\right.\\ \left.+\mathrm{Tr}\left({\left(X+\mathrm{GW}\right)}^{T}d\left(P^{T}1\right)\left(X+\mathrm{GW}\right)\right)\right\}\\ +\frac{D\sum_{j=1}^{M}\sum_{i=1}^{N}q(z_{ji})}{2\sigma^{2}}\\ =0 \end{array}$$

We have:

(19) $$\begin{array}{c} \sigma^{2}=\frac{1}{D\sum_{j=1}^{M}\sum_{i=1}^{N}q(z_{ji})}\left\{\mathrm{Tr}\left(Yd(P1)Y\right)\right.\\ -2\mathrm{Tr}\left({\left(P^{T}Y\right)}^{T}\left(X+\mathrm{GW}\right)\right)\\ \left.+\mathrm{Tr}\left({\left(X+\mathrm{GW}\right)}^{T}d\left(P^{T}1\right)\left(X+\mathrm{GW}\right)\right)\right\} \end{array}$$

Similarly,

(20) $$\begin{array}{c} \frac{\partial E_{L}\left(\Theta ,\Theta^{\left(m\right)}\right)}{\partial W}\\ =\frac{1}{2\sigma^{2}}\left(-2GP^{T}Y+2Gd\left(P^{T}1\right)X+2Gd\left(P^{T}1\right)\mathrm{GW}\right)\\ +2\mathrm{GW}+2\beta \mathrm{GBX}+2\beta \mathrm{GBGW}\\ +2\gamma GS^{T}d\left(P^{T}1\right)\mathrm{SGW}\\ =0 \end{array}$$

W can be obtained by solving the following system

(21) $$\begin{array}{c} \left(d\left(P^{T}1\right)\mathrm{GW}+2\sigma^{2}I+2\sigma^{2}\beta \mathrm{BG}+2\sigma^{2}\gamma S^{T}d\left(P^{T}1\right)\mathrm{SG}\right)W\\ =P^{T}Y-d\left(P^{T}1\right)X-2\sigma^{2}\beta \mathrm{BX} \end{array}$$

The proposed feature-based image registration algorithm is summarized in Algorithm 1.

**Algorithm 1:** The proposed non-rigid feature-based image registration algorithm

**Require:**

   The feature point $X={\left\{x_{i}\left|x_{i}\in {ℜ}^{D}\right.\right\}}_{i=1}^{N}$ and $Y={\left\{y_{j}\left|y_{j}\in {ℜ}^{D}\right.\right\}}_{j=1}^{M}$, parameters *w*, α, β, and γ.
  1:Initialize W=0, $P=I_{N\times N}$
  2:Initialize W=0, $P=I_{N\times N}$
  3:Search the *K* nearest neighbors for each point in X.  4:Perform L by minimizing the Equation (9)  5:Compute ${\left\{h_{ij}\right\}}_{i=1:N}^{j=1:N}$ as Equation (13)  6:**while** converged **do**  7:E-step:  8:   Extract the local shape to assign the membership probability $\pi_{ji}$  9:   Update the $q(z_{ji})$ as Equation (16)10:M-step:11:   Update $\sigma^{2}$ as Equation (19)12:   Update W by solving the linear system as Equation (21)13:
**end while**


**Ensure:**

   The aligned point set is f(X)=X+GW
The probability of correspondence is given by P


## 4. Performance Validation

In this section, experiments are carried out to evaluate the performance of the proposed method. The state-of-the-art algorithms ICP method http://www.cvlibs.net/software/libicp/ [14,30], TPS-RPM method https://www.cise.ufl.edu/~anand/publications.html [16], CPD method https://sites.google.com/site/myronenko/research/cpd [18], and CPD-GL method https://sites.google.com/site/jiayima2013/ [31] are used for comparison. The criteria for performance evaluation is chosen as

(22) $$error=\sqrt{\frac{1}{\left|S\right|}\sum_{(n,m)\in S}{∥f\left(x_{n}\right)-y_{m}∥}^{2}}$$

where *S* is the true matches between the two point sets. The error refers to the registration error.

### 4.1. Parameter Settings

**Figure 1:**
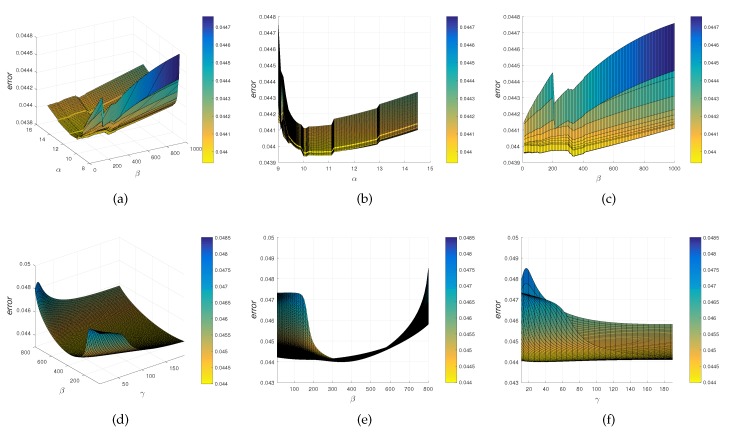
Model selection of the regularization parameters α, β, and γ for structure constraints. (**a**) model selection of the regularization parameters α and β, (**b**) and (**c**) are the different perspectives of (**a**), (**d**) model selection of the regularization parameters β and γ, (**e**) and (**f**) are different perspectives of (**d**).

In the proposed method, there are seven parameters: *w*, τ, *K*, ε, α, β, and γ. Parameter *w* is a weight of the uniform distribution for noise and outlier. Parameter τ is the width of the Gaussian kernel for non-rigid transformation function. Parameter *K* is the number of neighbors used to perform the matrix L in Equation (9) for the first local structure descriptor. Parameter ε is a threshold used to construct the neighborhood graph for the second local structure descriptor. Parameters α, β, and γ are three trade-off parameters for global and local constraints. The other four parameters *w*, τ, *K*, ε are selected as [31]. The model selection is shown in Figure 1. In this experiment, the fish dataset is performed and the registration error error is chosen as the metric. It is observed that the proposed method has almost the same performance when α∈[9,11], β∈[200,400], and γ∈[20,40]. Therefore, we set *w* = 0.1, τ = 2, *K* = 5, ε = 0.05, α = 10, β =340, and γ = 24.

### 4.2. Synthesized Point Set Registration

**Figure 2:**
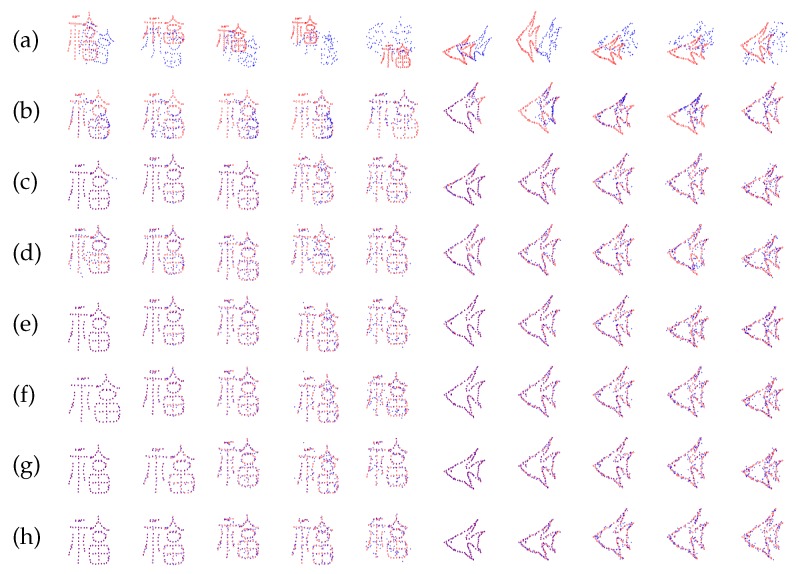
Registration results of point set registration algorithms on the fish and Chinese data with vary noise degradation. (**a**) The data with different gradation level; (**b**) iterative closest point (ICP); (**c**) thin-plate spline (TPS)-RPM; (**d**) CPD; (**e**) CPD-GL; (**f**) the proposed method with β=0 and γ=0; (**g**) the proposed method with β=340 and γ=0; (**h**) the proposed method with β=340 and γ=24.

**Figure 3:**
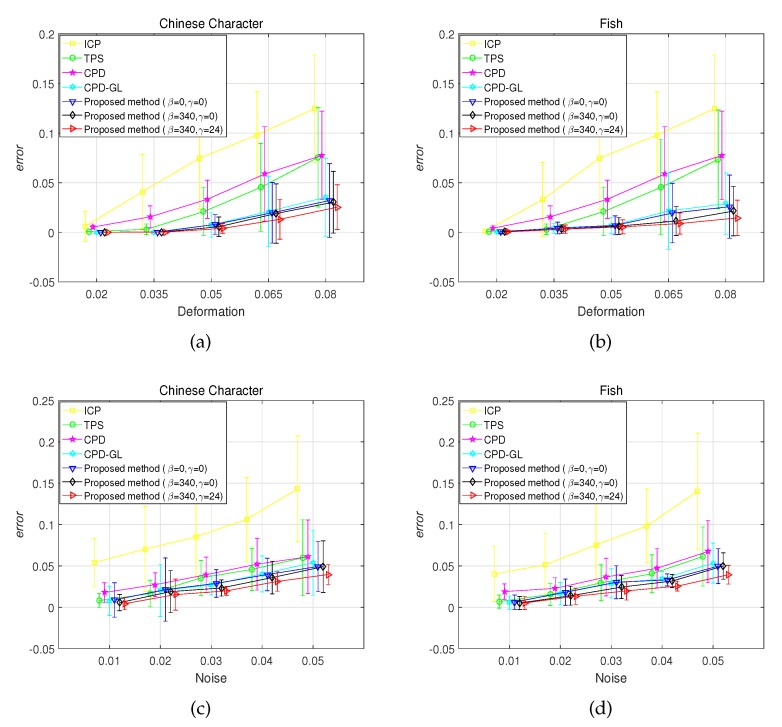
Registration error on Chinese character and fish shape under vary deformation and noise degradation. Sub-figures (**a**) and (**b**) show the registration error on Chinese character and fish shape under vary deformation, respectively, Sub-figures (**c**), and (**d**) show the registration error on Chinese character and fish shape under vary noise degradation, respectively.

**Figure 4:**
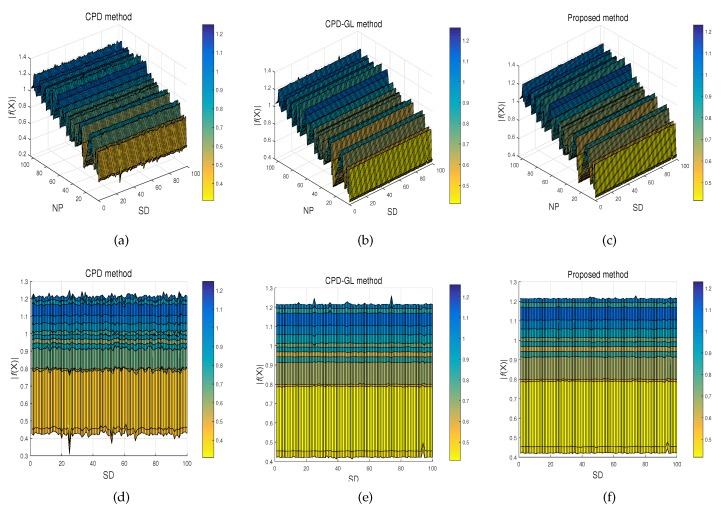
The performances of smoothness of the transformation by CPD, CPD-GL, and the proposed methods. Sub-figures (**a**), (**b**), and (**c**) show the performances of smoothness of the transformation using CPD, CPD-GL, and the proposed methods, respectively, while (**d**), (**e**), and (**f**) depict the same figures in another perspective.

This test is based on the synthesized dataset, which is constructed by Chui and Rangarajan [16]. It consists of two point sets: Chinese character and fish shape. For each model, we designed five sets of data to measure the robustness of registration algorithms with respect to different degrees of deformation and noise. In the deformation test, we use Gaussian radial basis functions to generate deformations, where the coefficients are sampled from a Gaussian distribution with zero mean and standard deviation ranging from 0.02 to 0.08. For the noise test, the standard deviation of the Gaussian noise added to the the original data ratio ranges from 0.01 to 0.05. In each test, one of the above distortions is applied to the model set to create an observed data set, and 100 samples are generated for each degradation level. Comparisons on the fish and Chinese data with varying noise degradation are shown in Figure 2. It is observed that the proposed method with β=0 and γ=0 has almost the same performance as the CPD-GL method, and the proposed method with β=340 and γ=0 can register the point set under different degradation slightly better than CPD-GL, whereas the proposed method with β=340 and γ=24 has the best performance. The metrics under different noise and deformation in fish and Chinese dataset are shown in Figure 3. Apparently, the proposed method with β=340 and γ=24 has the best performance among all. Furthermore, in order to show the smoothness of the transformation using the proposed method, the experiment of the standard deviation 0.03 for the Gaussian noise added to the Chinese data is performed and 100 samples are generated. The performances of smoothness of the transformation by CPD, CPD-GL, and the proposed method are given in Figure 4, where NP and SD denote the points in the Chinese dataset and the number of samples, respectively, |f(X)| is defined as $\left|f\left(X\right)\right|=\sqrt{f_{x}{\left(X\right)}^{2}+f_{y}{\left(X\right)}^{2}}$, $f_{x}\left(X\right)$ and $f_{y}\left(X\right)$ denote the 1st and 2nd dimensions of f(X), respectively. As can be observed, the proposed method achieves smoother performance than other methods.

### 4.3. IMM Hand Landmark Registration

**Figure 5:**
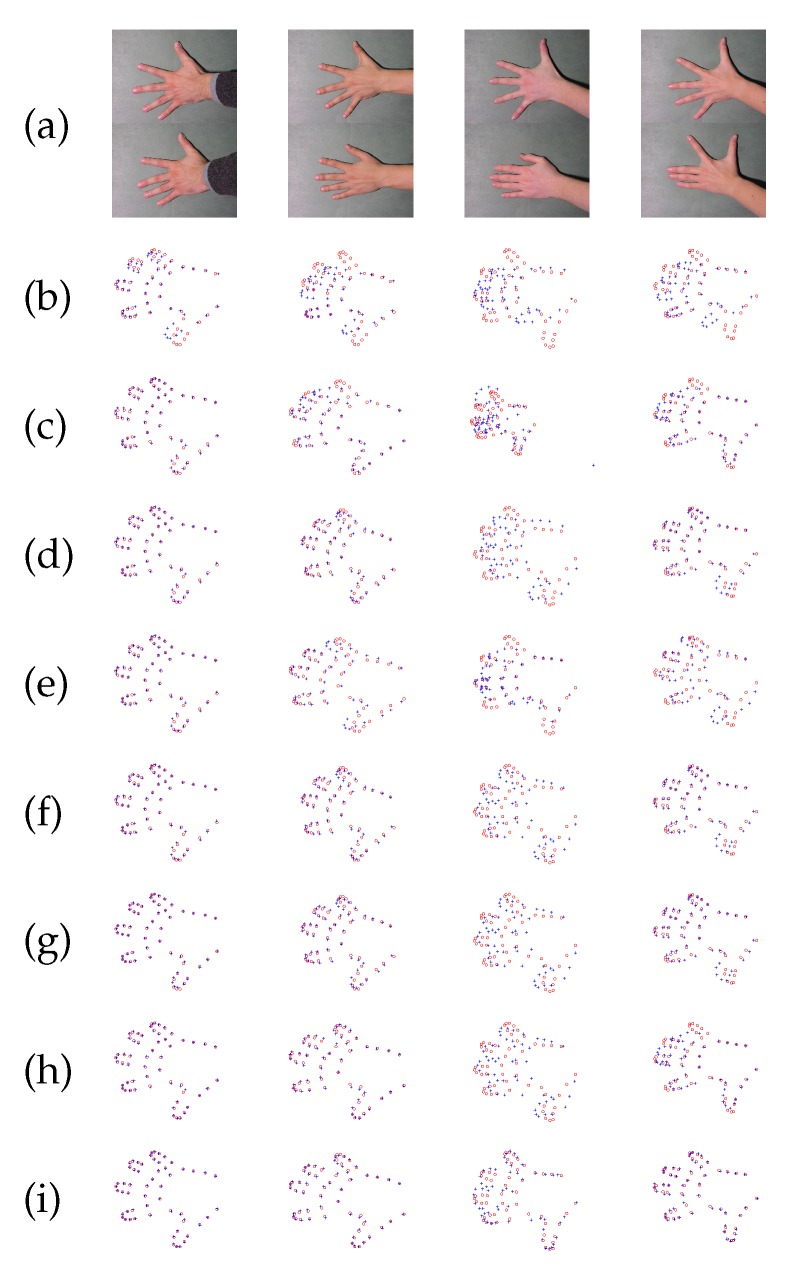
Four examples of IMM hand landmark registration results for each group. (**a**) four hand landmark images (**b**) target hand shape for each group (**c**) ICP (**d**) TPS-RPM (**e**) CPD (**f**) CPD-GL (**g**) the proposed method with β=0 and γ=0 (**h**) the proposed method with β=340 and γ=0 (**i**) the proposed method with β=340 and γ=24.

**Figure 6:**
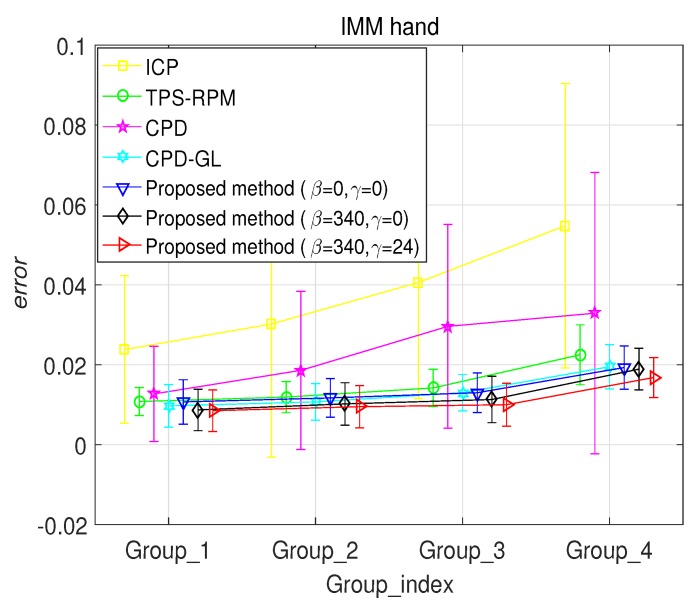
Registration error on IMM hand landmark.

A hand landmark experiment is used to evaluate the performance of the proposed method. The benchmark IMM Hand Database http://www2.imm.dtu.dk/pubdb/views/publication_details.php?id=403 is used. It consists of 40 images of 4 groups of the different human left hand. Images were acquired with resolution 800×600 JPG format in December 2001. Each group has 10 samples with different poses. The hand structures in the dataset have 56 landmarks with the ground truth correspondences. In this experiment, we use 4 groups for evaluation. For each group, the first hand shape is used as the model point set, another 9 hand shapes are considered as data point sets. Therefore, the ground truth correspondences of nine hand landmark pairs for each group are constructed. The proposed method is used to align these face landmark pairs. Comparisons are given in Figure 5. It is observed that the proposed method with β=340 and γ=24 outperforms than the other methods. Then, comparing the registration error are given in Figure 6, which the performance of the proposed method with β=340 and γ=24 has the best performance.

With respect to synthesized point set and IMM hand landmark, CPD-GL outperforms ICP, CPD, and TPS, whereas the proposed method with β=0 and γ=0 has the almost same performance with CPD-GL method. Moreover, the proposed method with β=340 and γ=0 has slightly better performance than the CPD-GL method, and the proposed method with β=340 and γ=24 has the best performance. Then, the computational complexity of the proposed method is analyzed. The computational complexity for searching the *K* nearest neighbors for each local track in X needs O((K+N)logN) by using the k−d tree, the computational complexity for performing the matrix L needs $O(K^{3}N)$, the EM algorithm needs almost $O(N^{3})$. Therefore, the total computational complexity of the proposed algorithm is $O(N^{3})$. All algorithms are implemented in MATLAB, and the tests are performed on a Core i7-4790 3.6GHz with 8GB RAM. The run-times of these algorithms are given in Table 1, which illustrates that the proposed method has almost the same computation time with CPD-GL method.

### 4.4. 4DCT_75 Dataset

**Figure 7:**
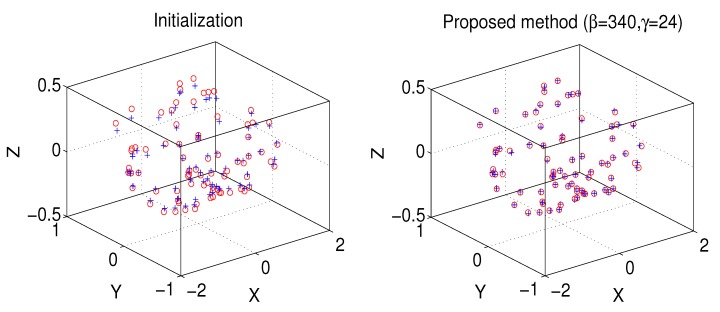
A example of the registration result of the 4DCT_75 data, while the left column denotes the initialization, the right column denotes the registration result using the proposed method with β=340 and γ=24.

**Figure 8:**
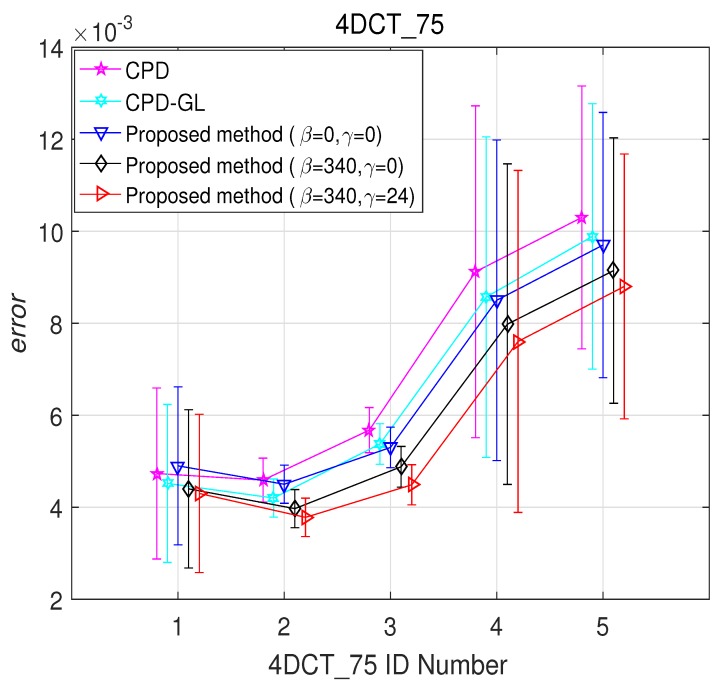
Registration error on “4DCT_75”.

The 4DCT_75 dataset of the benchmark data from the DIR-lab http://www.dir-lab.com/index.html is used. The 4DCT_75 data contains ten (“case1” to “case10”) thoracic 4D CT images. Each 4D CT images have ten 3D CT images. In particular, we chose “4DCT_75_T00” as a model point set. The other five point sets (“4DCT_75_T10” to “4DCT_75_T50”) are set as the scene point sets. Thus, we have 50 pairs of point sets. The example of the registration result of the proposed method are shown in Figure 7, which the proposed method with β=340 and γ=24 can align these point pairs. Furthermore, the registration errors of CPD method, CPD-GL method, and the proposed method are shown in Figure 8. It is shown that the proposed method with β=340 and γ=24 has a better performance than the other methods.

### 4.5. Real Image Feature Matching

**Figure 9:**
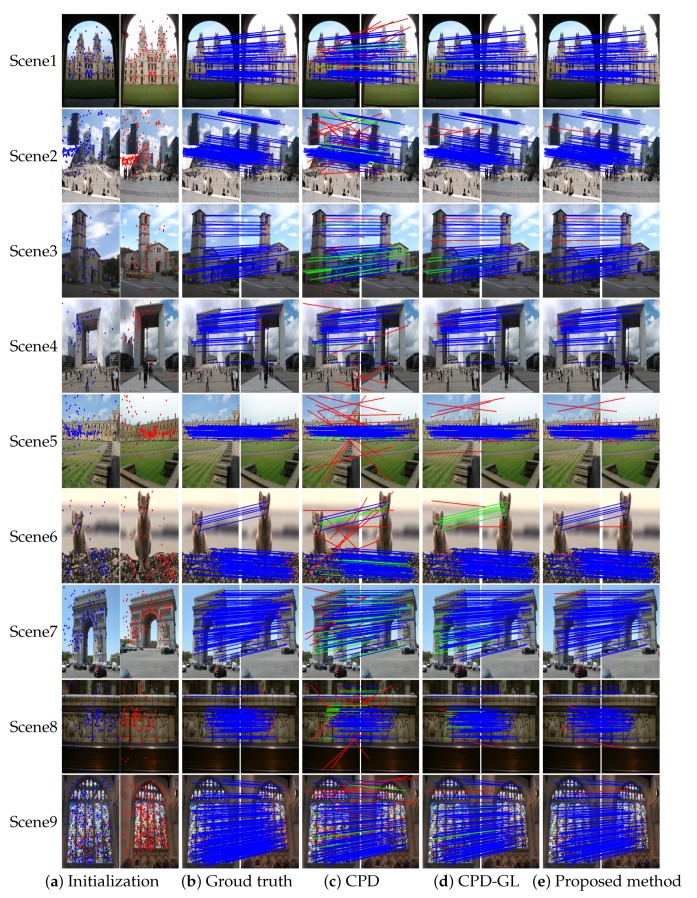
Different scenes on real image data. (**a**) different image pairs extracted feature points from scale-invariant Fourier transform (SIFT) algorithm (**b**) manual real correspondence, with results from (**c**) CPD method, (**d**) CPD-GL method, (**e**) proposed method. Each line segment corresponds to the position of the corresponding feature point in the two images (blue = true positive, green = false negative, and red = false positive). For visibility, 100 pairs are selected for some pair of images as presented, and the true negatives are not shown.

**Figure 10:**
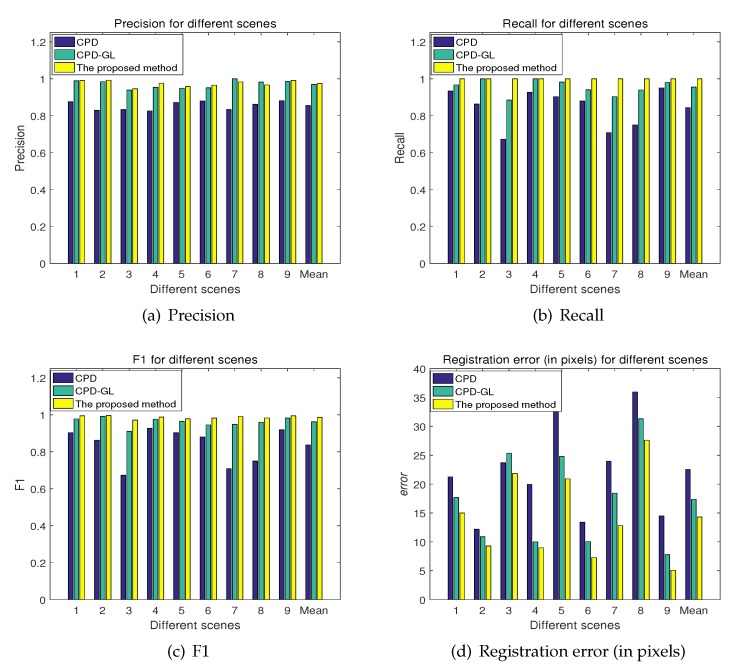
The results of different evaluation metrics of the CPD method, CPD-GL method, and the proposed method in different scenes. (**a**) Precision (**b**) Recall (**c**) F1 (**d**) Registration error (in pixels).

In this experiments, the real image pairs are obtained from the data of Jiayi Ma [31], Tuytelaars [32], the Oxford buildings dataset http://www.robots.ox.ac.uk/~vgg/data/oxbuildings, and the Paris dataset http://www.robots.ox.ac.uk/~vgg/data/parisbuildings. The SIFT algorithm is used to extract the feature points of each image, and the putative correspondence is created by nearest neighbor matching. The goal is to find correspondences/matches between two sets of feature points. The performance is evaluated by precision, recall, and F1, where the F1 measure is used to evaluate the balance between recall and precision. The precision, recall, and F1 are defined by: $\mathrm{Precision}=\frac{N_{L}}{N_{C}}$, $\mathrm{Recall}=\frac{N_{L}}{N_{P}}$, and $F1=\frac{2\cdot \mathrm{Recall}\cdot \mathrm{Precision}}{\mathrm{Recall}+\mathrm{Precision}}$, where $N_{L}$, $N_{C}$, and $N_{P}$ denote the preserved inlier number, the preserved correspondence number, and the inlier number contained in the putative correspondences, respectively. The ground truth is established in advance manually. The results of matching these image pairs are illustrated in Figure 9. It is observed that the proposed method can distinguish inliers from the outliers. Furthermore, the performance of CPD, CPD-GL and the proposed method in different scenarios is shown in Figure 10. It is observed that the proposed method achieves slightly better performance in precision, recall and F1 metrics compared to other methods. Furthermore, the registration error error in pixels is proposed to evaluate the performance of these algorithms. Figure 10d demonstrates that the proposed method performs better than the CPD method and CPD-GL method. Based on the above evaluation results, the proposed method has better performance than other methods.

## 5. Discussion

In this paper, the proposed method is compared with the CPD and CPD-GL methods. The CPD registration method only preserves the global structure and ignores the local structure of point set. Although the CPD-GL algorithm preserves the global and local structure, it only introduces one local structure descriptor. In the proposed method, two local structure descriptors are constructed to preserve the local structure of feature points. The first one is the connectivity matrix-based constraint, which retains the local neighborhood relationship, while the other is the Laplacian coordinate-based constraint, which encodes the local neighborhood scale. Therefore, the proposed method preserves the local structure in terms of the neighborhood relationship and neighborhood scale, and it is more flexible. To illustrate the advantages of this method, three experiments were performed based on the proposed method and compared with the state-of-the-art registration algorithms. In the first experiment, for the 2D point set registration, the experimental results show that the proposed algorithm outperforms other algorithms and has almost the same computation time with CPD-GL algorithm. In the second experiment, registration error is employed to estimate the proposed algorithm on the 3D data, and the proposed algorithm outperforms other algorithms. Furthermore, the proposed algorithm is tested on several image pairs under many different evaluation metrics. The performances of these registration algorithms are evaluated by precision, recall, F1, and registration error. From the above experimental results, the proposed method outperforms the other methods. In particular, it is better to use the proposed method which provides stable and accurate transformation estimation for handling complicated non-rigid deformation. When deformation is not significant, the CPD, CPD-GL, and the proposed method obtain almost similar results, and the CPD has lower computation complexity.

There is room for further development of the proposed framework, especially proposing a fast algorithm and developing more evaluation metrics for point set registration such as smoothness of the transformation. It is interesting to investigate some real-life scenarios, where point set correspondences are weaker such as clouds of points with different amounts of points, missing correspondences or points. Furthermore, the proposed point set registration framework can be applied to perform track-to-track association with sensor bias in distributed multi-target tracking [33].

## 6. Conclusions

In this paper, a GMM-based probabilistic method for image feature matching is proposed. The feature points of one image are represented by GMM centroids, and the feature points of the other image are fitted to the first image feature points by maximizing the likelihood. To preserve the local structure of these feature points, two local structure descriptors are constructed. The EM algorithm is used to perform image feature matching. Computer simulations confirm the effectiveness of the proposed method in registering different types of images compared to the state-of-the-art registration algorithms.

## Figures and Tables

**Table 1:** Runtime of these point set registration algorithms on different datasets.

Method	Fish	Chinese	IMM Hand
ICP	0.4896s	0.3343s	0.1283s
TPS	2.3457s	1.6755s	0.6231s
CPD	0.1853	0.1278s	0.0954s
CPD-GL	2.2667s	1.4249s	0.3449s
Proposed method	2.4665s	1.7751s	0.4624s
